# Fates of Emitted Particles Depending on Mask Wearing Using an Approach Validated Across Spatial Scales

**DOI:** 10.1002/gch2.202300008

**Published:** 2023-05-03

**Authors:** André Baumann, Dennis Hoch, Jennifer Niessner

**Affiliations:** ^1^ Institute for Flow in Additively Manufactured Porous Media (ISAPS) Heilbronn University of Applied Sciences Max‐Planck‐Straße 39 74081 Heilbronn Germany

**Keywords:** CFD simulation, face masks, particle fates, multi‐scale modeling, aerosol particle transport

## Abstract

The spread of emitted potentially virus‐laden aerosol particles is known to be highly dependent on whether a mask is worn by an infected person and on the emission scenario, i.e., whether the person is coughing, speaking, or breathing. The aim of this work is to investigate in detail the fates of particles emitted by a person wearing a perfectly fitting, a naturally fitted mask with leakage, and no mask depending on the emission scenario. Therefore, a two‐scale numerical workflow is proposed where parameters are carried through from a micro‐scale where the fibers of the mask filter medium and the aerosol particles are resolved to a macro‐scale and validated by comparison to experimental measurements of fractional filtration efficiency and pressure drop of the filter medium as well as pressure drop of the mask. It turns out that masks reduce the number of both emitted and inhaled particles significantly even with leakage. While without a mask, the person opposite of an infected person is generally at the highest risk of being infected, a mask worn by an infected person speaking or coughing will deflect the flow leading to the fact that the person behind the infected person might inhale the largest number of aerosol particles.

## Graphical abstract

The spread of emitted virus‐laden aerosol particles is highly dependent on whether a mask is worn and on the emission scenario, i.e., coughing, speaking, or simply breathing. This work aims at investigating in detail the fates of particles emitted by an infected person wearing a perfectly fitting, naturally fitted mask, and no mask depending on the emission scenario.

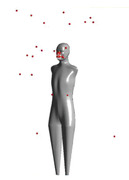

## 1 Introduction

The corona pandemic has been affecting society for a long time, and research on the transmission of the virus is going on. Experts agree on the fact that aerosol transmission plays a major role in spreading SARS‐CoV‐2 and other diseases, such as influenza or measles. Social distancing, both in indoor and outdoor environments as well as residence time are factors influencing the risk of infection. In addition, many flow parameters are decisive for the transport of the aerosol particles. Velocity of emitted particles, particle emission rate, particle size distribution, heat emissions of persons, or ambient flows are all aspects that have to be considered in order to predict and determine the dispersion of particles, and thus the risk of infection.

To investigate the influence of these parameters, CFD (Computational Fluid Dynamics) simulations represent an excellent option to describe the transport of human exhaled aerosols. Mirzaei et al. and Hossain and Faisal, for example, studied the exhalation and dispersion of a single person and investigated the distance that aerosol particles are transported.^[^ ^1^, ^2^ ^]^ Their studies show that aerosol clouds do not travel further than 1 m when talking or coughing and ≈0.2 m when breathing.^[^ ^2^ ^]^ Other simulations show that aerosol clouds can reach up to ≈2.7 m.^[^ ^1^ ^]^ Here, the velocity range reported is very broad. In both studies, the focus is on the exhalation process and the area directly behind the inlet (mouth) is resolved. On the contrary, external influences from the person (shape, body heat) or environment (e.g., wind) are not considered.

In Chea et al., Feng et al., Gao and Niu, and Blocken et al., it is evaluated how many particles can travel from one person to another.^[^ ^3^, ^4^, ^5^, ^6^ ^]^ All studies indicate that the proposed social distances may not be sufficient. Specifically, in Chea et al. particles are shown to be transported up to 2.77 m, ≈3 m are reported by Feng et al., and ≈10 m according to Blocken et al.^[^ ^3^, ^4^, ^6^ ^]^ Flows induced by wind or ventilation play a crucial factor in these regards. Chea et al. and Feng et al., for instance, illustrate that the influence of ambient flows from the environment (wind) significantly affects the distance particles are transported.^[^ ^3^, ^4^ ^]^ In Blocken et al., the influence of the velocity of two persons and their position on each other is investigated.^[^ ^6^ ^]^ Corzo et al. compared the infection risk in a bus for a motionless case to a case where mechanical ventilation is applied. It has been shown that in the motionless case, high probabilities of infection occur in the surrounding area of the emitter, and mechanical ventilation can reduce this risk down to only 3%, and down to <1% for an HVAC system supplying 25% of fresh air.^[^ ^7^ ^]^

There are also simulative studies on the effect of measures for reducing the risk of infection. Chen et al. found that covering a cough or turning the head away can prevent the receptor's direct exposure due to significantly reduced horizontal velocity.^[^ ^8^ ^]^ Dispersion in a room, as well as specific ventilation concepts, are studied in Motamendi et al. and Borro et al.^[^ ^9^, ^10^ ^]^ It was shown that a doubling of the flow of an HVAC system supplying conditioned fresh air allows for a relevant reduction of up to 77% in droplet mass concentration compared to a nominal airflow.^[^ ^9^ ^]^ In addition, Gao et al. pointed out that by ventilation the particle spread can be significantly reduced.^[^ ^5^ ^]^

The wearing of face masks is recommended or mandatory almost all over the world. Masks are an especially attractive protection measure since they provide both third‐party and self‐protection^[^ ^11^ ^]^. Bagheri et al., for example, compared the risk of infection of two facing individuals and found a significant reduction of infection risk from 90% when neither person wore a mask to only 0.4% when both persons wore a well‐fitting FFP2 mask.^[^ ^12^ ^]^ These results were calculated analytically for a distance of three meters between two speaking persons and an exposure duration of 20 min using a polypathogen model and a comprehensive database for fluid and particle parameters. Similarly, the previously mentioned studies Chea et al. and Feng et al. indicate that face masks significantly reduce the particle spread.^[^ ^3^, ^4^ ^]^

However, masks do not only provide a filtering effect for the inhaled and exhaled air but also generate a pressure drop. Finding a high filtration efficiency at a low pressure drop is a challenge. In many studies, a direct comparison is drawn between breathing without and with a mask.^[^ ^13^, ^14^, ^15^ ^]^ The effect of a mask on the flow profile of exhalation influences the risk of infection in different life situations e.g. in a restaurant or supermarket as well as in a bus or an airplane.^[^ ^16^, ^17^ ^]^ In these studies, only numerical modeling and simulation were applied, although experimental validation significantly increases the reliability of simulation in general. However, an experimental validation often requires a large effort and time expenditure and is therefore partly neglected. Thus, although there are many studies on mask‐wearing in these situations, the influence of mask‐wearing on particle transport is not thoroughly investigated.^[^ ^18^, ^19^, ^20^ ^]^ Leakage due to a bad fit of the mask may also influence particle transport significantly. Xi et al. found that a leakage area of only 0.5 cm^2^ can lead to a reduction of filter efficiency of 9%.^[^ ^21^ ^]^ Chiera et al. and Ortiz determined that also the shape, type, and design of the mask has a significant influence on filtration efficiency.^[^ ^22^, ^23^ ^]^ Ortiz et al. visualized different escape routes from masks using colored water mist.^[^ ^23^ ^]^ In addition, CFD simulation was used for the optimization of a face mask.^[^ ^24^ ^]^ In all these studies the parameters of the masks are not determined based on the “real” fibrous geometry of the masks filter medium obtained e.g. by microCT scans along with micro‐scale simulations, but values for pressure drop and filtration efficiency are partly assumed. This means, that parameters have not been carried through across scales yet so the fibrous structure of the mask material has not been accounted for so far.

**Figure 1:**
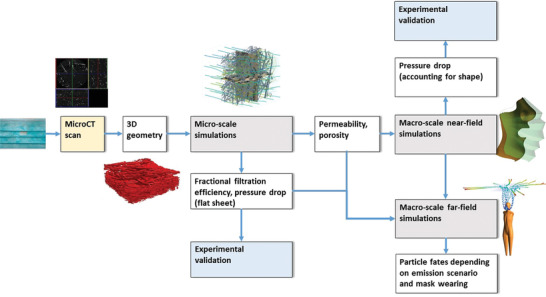
Scheme and procedure for simulations (grey) and results (white) from a section of a mask to micro‐ and macro‐CFD simulations.

Therefore, in this study, fates of emitted particles when exhaling, speaking, and coughing with a mask (both naturally fitted and perfectly sealed) and without a mask are investigated. For this purpose, the geometry of existing fibrous mask materials is analyzed by means of microCT scans and evaluated by micro‐scale simulations for fractional filtration efficiency, see **Figure** **1**. The data obtained is then transferred to the macro‐scale where the porous structure is not resolved anymore, but described by averaged parameters, such as porosity and permeability. This allows to determine and validate the pressure drop of the mask accounting for shape. Finally, the far‐field protection effect of masks will be examined in detail. Specifically, the particle fates of a person wearing a perfectly fitting, natural fit and no mask will be compared for the emission scenarios of breathing, speaking, and coughing.

Although face masks generate further global challenges besides infection prevention, such as environmentally friendly manufacturing^[^ ^25^ ^]^ and disposal, this study is restricted to the technical aspects of face masks as a device for preventing the spread of particles. While the production and disposal of face masks remain critical challenges in the context of public health and sustainability, our study aims to advance the understanding of the physical behavior of particles and the impact of mask material properties on their transport, filtration efficiency, and pressure drop.

This paper is organized as follows:

In Section 2, we present the theory and basic equations used in the CFD simulations. Next, in Section 3, we present the setup of our numerical systems including geometry, how parameters are determined based on microCT scans as well as properties of particles and environmental conditions. Section 4 is for presenting and discussing simulation results while in Section 5, we sum up and give an outlook on future work.

## 2 Theory and Basic Equations

For the description of the flow of the continuous (air) phase, the conservation equations for mass, momentum, and energy are used in an Eulerian setting. Subsequently, the integrated force equations describing aerosol particle transport in a Lagrangian reference frame are presented.

For the solution of the set of flow and transport equations, ANSYS Fluent, version 2020R2, was used.

Mass conservation of the air phase is given by:

(1) $$\begin{array}{c} \frac{\partial \rho }{\partial t}+\nabla \cdot \;\left(\rho \vec{v}\right)=\;0 \end{array}$$

where *v* is the fluid velocity vector and *ρ* the density. The conservation of air‐phase momentum is described by:

(2) $$\begin{array}{c} \frac{\partial \left(\rho \vec{v}\right)}{\partial t}+\nabla \cdot \;\left(\rho \vec{v}\vec{v}\right)=\;-\nabla p+\rho \vec{g}+\nabla \cdot \bar{\bar{\tau }} \end{array}$$

with *p* as the static pressure, *ρg* as the gravitational body force and the stress tensor

(3) $$\begin{array}{c} \bar{\bar{\tau }}=\;\mu \left[\left(\nabla \vec{v}+\nabla {\vec{v}}^{T}\right)-\frac{2}{3}\nabla \cdot \vec{v}I\right] \end{array}$$

where *µ* is the dynamic viscosity and *I* the unit tensor. Finally, the conservation of energy of air is given by

(4) $$\begin{array}{ccc} & & \frac{\partial \left(\rho E\right)}{\partial t}+\nabla \cdot \left[\vec{v}\left(\rho E+p\right)\right]\\ & & \;=\nabla \cdot \left[\left(k_{\mathrm{eff}}\nabla T\right)-\sum_{i}h_{j}\vec{J_{j}}+\left(\bar{\bar{\tau_{\mathrm{eff}}}}\cdot \vec{v}\right)\right] \end{array}$$

where *k_eff_* is effective conductivity, *J_J_* is the diffusion flux of species *j*, and *h* is the enthalpy.

Particle transport in the Lagrangian framework is governed by

(5) $$\begin{array}{c} \frac{du_{p}}{dt}=F_{D}\;\left(v-v_{p}\right)+\frac{g_{x}\left(\rho_{p}-\rho \right)}{\rho_{p}}+F_{x} \end{array}$$

where *v_p_* is the velocity of the injected particles and *ρ_p_* the density of the particles. *F_x_* represents an additional (force/unit particle mass) acceleration term which is related to the Saffman lift force in our case. The term *F_D_(v‐v_p_)* is the drag force per unit particle mass with

(6) $$\begin{array}{c} F_{D}=\frac{18\mu }{\rho_{p}d_{p}^{2}}\;\frac{C_{D}}{24}Re \end{array}$$

where *C_D_* is the drag coefficient and *d_p_* is the particle diameter. *Re* represents the relative Reynolds number defined by

(7) $$\begin{array}{c} Re\equiv \frac{\rho d_{p}\left|v_{p}-v\right|}{\mu } \end{array}$$

## 3 Workflow and Numerical Setup

Starting with microCT scans of a mask and the reconstruction of the 3D geometry (Section 3.1), the setup of the micro‐scale simulations is described in Section 3.2, where fibers and particles are resolved, see Figure 1. The result of these simulations is the fractional filtration efficiency, the pressure drop, and macro‐scale material parameters. Using macro‐scale simulations, the pressure drop accounting for the shape of the mask (near‐field consideration) is modeled and validated in Section 3.3. In Section 3.4, the far‐field effects of mask‐wearing, i.e. the particle fates, are studied depending on the emission scenario and the compliance of mask‐wearing. Section 3.5 then considers the macro‐scale simulation setup for inhalation.

### 3.1 MicroCT Scans and Reconstruction of the 3D Geometry

**Figure 2:**
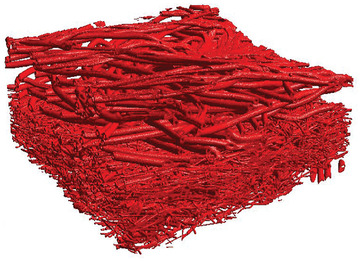
Voxelized 3D geometry of the fibrous structure of one supporting layer (top, coarse fibers) and the filter medium (fine fibers) of a surgical mask obtained from a microCT scan.

The 3D geometry of one supporting layer as well as the filter layer of a mask is reconstructed using a stack of 2D images obtained by microCT scans. The resolution of the scan was set to 0.4 µm per pixel. The scanned section was 1.6 × 0.94 × 0.49 mm^3^ in total. An example of a scanned voxelized 3D geometry is shown in **Figure** **2**. Details of the procedure are given in Hoch et al.^[^ ^26^ ^]^

### 3.2 Micro‐Scale Numerical Setup

Based on the reconstructed voxelized 3D geometry of the fibrous structure, micro‐scale Eulerian‐Lagrangian simulations are carried through for determining fractional filtration efficiency, pressure drop as well as porosity and permeability needed as input to the macro‐scale simulations, see Figure 1. The flow enters the domain at the top with a velocity of 0.117 m s^−1^ (which corresponds to a volume flow of 95 l min^−1^ through the respective area as defined in DIN EN 149) and leaves through a pressure outlet at the bottom. All sides are periodic boundaries and the fibers are considered impermeable. For the determination of the filtration efficiency, a particle size distribution with diameters ranging from 0.1 to 10 µm is injected (1000 particles per diameter).

### 3.3 Near‐Field Macro‐Scale Simulation

**Figure 3:**
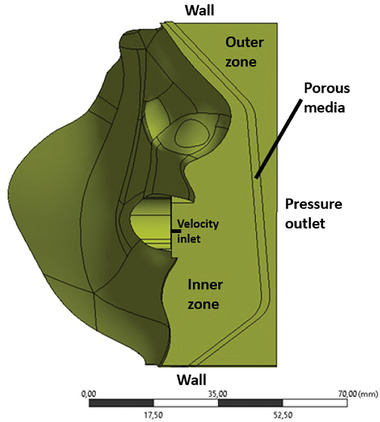
Geometry, boundary conditions, and zones are used for setting up the macro‐scale near‐field simulations exemplarily for the case of an FFP2 mask.

**Figure 4:**
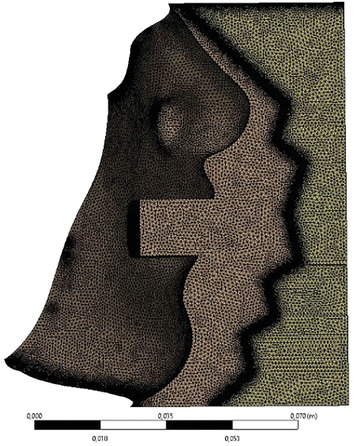
Mesh used in the macro‐scale near‐field simulations using a surgical mask. The thickness of the mask is resolved using 8 cells.

Using the permeability obtained by the micro‐scale simulation the pressure loss of an entire mask is determined by accounting for its shape. For this purpose, an FFP2 and a surgical mask are constructed and adapted to a standard head according to Berger et al.^[^ ^27^ ^]^ Like this, the no‐leakage case is described where the entire exhaled flux of 95 l min^−1^ is going through the mask.^[^ ^28^ ^]^ Because the ratio of thickness to the height of the mask is very small, a large number of elements (see **Table** **1**) is necessary to capture the velocity profile and the pressure drop accurately enough. For symmetry reasons, only half of the mask and face are considered. In order to reduce the influence of the boundary on the flow within the mask, the outer boundary is defined at a distance of a few centimeters from the mask. In **Figure** **3**, the numerical setup is shown exemplarily using an FFP2 mask, while the mesh is shown in **Figure** **4** using a surgical mask. The number of elements for both cases is shown in Table 1.

**Table 1:** Number of mesh elements for the macro‐scale near‐field simulations using surgical and FFP2 mask

Mask	Number of elements [‐]
Surgical mask	46 161 059
FFP2 mask	7 225 537

The solution domain is split into three different zones as shown in Figure 3. The “inner zone” is defined as the space between the face and the mask, while the “porous zone” consists of the mask itself. The volume outside the mask is called the “outer zone”.

At the mouth boundary surface, a constant influx velocity of 0.117 m s^−1^ is given while zero pressure is assumed at the outer boundary. The turbulence in “inner” and “outer” zone is modeled using the standard k‐epsilon model while flowing through the porous area is laminar and creeping (Re < 1). In order to investigate the average pressure drop during exhalation, flow is considered steady. The system of air‐phase conservation equations is solved by a phase‐coupled SIMPLE scheme. For space discretization, a least squares cell‐based method is used with second‐order for the pressure and momentum balance equations and first‐order upwinding for the discretization of the turbulent kinetic energy and specific dissipation rate. The first‐order upwinding methods were necessary to reduce/prevent major backflow. All simulations were carried through using default settings for the under‐relaxations factors and were performed using two Intel Xeon Gold 6126 and 24 logical processors.

### 3.4 Far‐Field Macro‐Scale Simulation – Emission

#### 3.4.1 Geometrical setup

**Figure 5:**
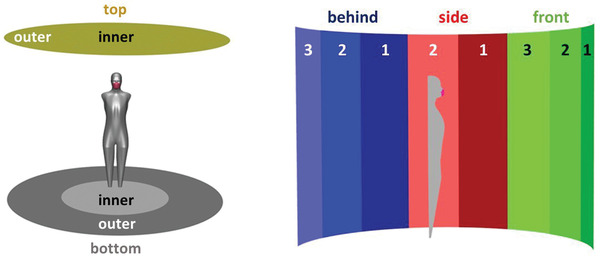
Geometrical setup of the far‐field macro‐scale simulations showing the thermal manikin and the top (yellow) and bottom (grey) boundaries on the left. Side boundaries split into three zones (front (green), side (red), behind (blue)) with two to three subzones each and thermal manikin used in the macro‐scale far‐field simulations on the right.

In the far‐field simulations, the effect of mask wearing and emission scenarios (exhaling through the nose or mouth, speaking, coughing) on the transport of aerosol particles to a potential host is investigated. Therefore, a simplified human body in an indoor space is considered and the transport of emitted particles up to a distance of 1.5 m from the central axis through the manikin is investigated, see **Figure** **5**. The cylindrical domain has a height of 2.25 m and the height of the person is 1.75 m.

As for the macro‐scale near‐field simulations, only half of the domain is considered for symmetry reasons. The room itself is open on the sides (zero pressure and temperature of 20.15 °C) and the top and bottom boundary are walls. In order to be able to investigate where the aerosol particles are transported, the side boundary is divided into eight equally sized zones (see Figure 5). In addition, the top and bottom boundaries are divided into a near‐field (radius < 0.75 m) and far‐field (0.75 m ≤ radius < 1.5 m) region.

#### 3.4.2 Meshing

The meshing was done with tetrahedrons using three prism layers at wall boundaries. In order to limit the overall number of elements, the mask is not modeled as a 3D body, but as a 2D porous jump condition. This has the advantage that no complex meshing has to be carried out throughout the thickness of the mask and the overall number of elements is moderate (12,388,291). The simulations shown in the following were carried through exemplarily using the FFP2 mask.

#### 3.4.3 Numerical Setup

Four different emission scenarios are considered: exhalation through the mouth and nose, talking, and coughing. For these situations, the mouth surface (for nasal breathing naturally the surface of the nose) is used as influx boundary where a constant temperature of 34 °C is given and a constant velocity is defined as given in **Table** **2**.^[^ ^28^ ^]^ These velocity values correspond to a volumetric flux of 30 l min^−1^ as defined by DIN EN 149. Additionally, the inhalation process is considered where a negative velocity is given at the influx boundary (nose). The body is modeled as thermal manikin emitting a heat rate of 744 W m^−^^3^.^[^ ^30^ ^]^ Further parameters are given in Table 2.

**Table 2:** Boundary conditions at the inlet and parameter values used in the macro‐scale far‐field simulations

Parameter	Value	Source
Velocity exhaling through nose [m s^−1^]	3.50	[26]
Velocity exhaling through mouth [m s^−1^]	0.76	[26]
Velocity speaking [m s^−1^]	4.07 (male)	[31]
Velocity coughing [m s^−1^]	15.30 (male)	[31]
Density person [kg m^−3^]	1066	[32]
Specific heat person [J kg^‐1^ K^‐1^]	3475	[32]
Thermal conductivity person [W m^−1^ K^−1^]	0.30	[32]

For the porous jump condition, the SIMPLE scheme and the standard pressure discretization are used. For spatial discretization of turbulence and energy, first‐order upwinding is used (again because of possible backflow). The momentum conservation equations are solved using the second‐order upwinding method. The relaxation factors are set to the default value in ANSYS Fluent, only that for momentum was reduced to 0.5.

#### 3.4.4 Setup of Particle Transport

After reaching steady‐state, the particles are injected using the discrete particle model (DPM) implying that also for aerosol particle transport, a steady‐state consideration is applied. This is consistent with the findings of Bulinska and Bulinski who showed that the particle transport is not strongly influenced by the breathing cycle.^[^ ^29^ ^]^ These injections are performed per every 2D facet of the inlet boundary and the particles are tracked until they hit a boundary where they are counted. The number of exhaled particles during 15 min is defined for each emission scenario as given by Hartmann et al.^[^ ^33^ ^]^

Evaporation and condensation are neglected since previous studies^[^ ^4^ ^]^ have shown that for the relevant aerosol particle sizes, the water component evaporates in < 1s. Particles are assumed to stick to walls. According to Fabian et al., Papineni and Rhosental, and Ding et al. more than 80% of particles emitted while breathing and talking are less than 1 µm in diameter.^[^ ^34^, ^35^, ^36^ ^]^ Following Hartmann et al. and Ding et al., four different diameters were selected to represent the particle size distributions as shown in **Tables** **3** and **4**.^[^ ^33^, ^36^ ^]^

**Table 3:** Ratio of injected particles for the emission scenarios “exhaling” and ”speaking”

Particle diameter [µm]	Ratio of injected particles [%]
0.3	35
0.5	35
0.72	20
1	10

**Table 4:** Number of injected particles for the emission scenarios “exhaling” and ”speaking”

Diameter [µm]	Exhaling through nose [‐]	Exhaling through mouth [‐]	Speaking [‐]
0.3	3,752	21,765	30,471
0.5	3,752	21,765	30,741
0.72	2,144	11,608	17,412
1	1,072	5,804	8,706

For coughing, the particle size distribution given in literature differs strongly as shown for example in Gralton et al.^[^ ^37^ ^]^ Following Lindsley et al., the distribution used in our study is chosen according to **Table** **5**.^[^ ^38^ ^]^

**Table 5:** Relative and absolute number of injected particles per diameter used in the emission scenario “coughing”

Diameter [µm]	Percentage [%]	Number [‐]
0.3	20	1,451
0.5	20	1,451
0.72	20	1,451
1	20	1,451
5	10	726
10	10	726

The properties and models applied to the particles are listed in **Table** **6**.

**Table 6:** Settings for particles for the DPM model

Temperature [°C]	Velocity	Drag law	Cunningham correction	Brownian motion	Discrete random walk model	Saffman lift force
34	Like velocity inlet for breathing, speaking or coughing	Stokes – Cunningham	1.1	Yes	Yes	Yes

#### 3.4.5 Setup of the Mask

In order to describe the filtration of the particles by the mask, a so‐called User Defined Function (UDF) is applied assigning a random number between 0 and 1 to each particle. Particles having a random number smaller than the fractional filtration efficiency are removed. If, for example, the fractional filtration efficiency for a given particle size is 0.9 and a particle of that size associated with a random number of 0.8 hits the mask it will be removed. The fractional filtration efficiency is given as determined from measurements by Berger et al. for a typical FFP2 mask, see **Table** **7**.^[^ ^27^ ^]^ Since no measurement data were available for particle diameters larger than 2 µm, a filtration efficiency of 100% is assumed.

**Table 7:** Fractional filtration efficiency used in the macro‐scale far‐field simulations according to Berger et al. for a size range of 0.3‐1 µm and assumption (5 and 10 µm).^[^ ^27^ ^]^

Diameter [µm]	Filter efficiency [%]
0.3	97.43
0.5	97.53
0.72	97.44
1	98.04
5	100
10	100

**Figure 6:**
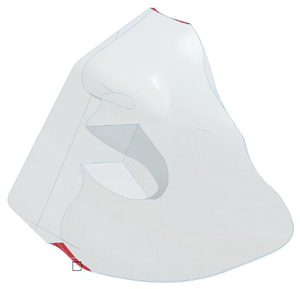
FFP2 mask with highlighted leakage areas in red for the far‐field simulations.

In order to investigate the influence of leakage on particle fates, slots of a total area of 1 cm^2^ at the top and bottom of the mask were selected as leakage areas as shown in **Figure** **6** (red surfaces).

This leakage area corresponds to ≈0.7% of the total area of the mask and is lower than the area reported in Xi et al. which means that the worn mask provides a good fit to the face.^[^ ^21^ ^]^

### 3.5 Far‐Field Macro‐Scale Simulation – Inhalation

**Figure 7:**
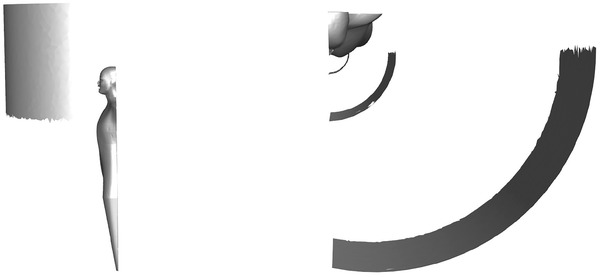
Planes at different distances from the inhaling person used as particle injectors.

In order to study the influence of mask‐wearing during inhalation, the same geometrical setup, numerical setup, and meshing are used as described in Section 3.3. Contrary to the emission scenarios, the injection of the particles start from planes in distances of 0.07, 0.225, and 0.725 m from the person at an initial velocity of 0 m s^−1^ (see **Figure** **7**).

## 4 Results and Discussion

In this section, the results of micro‐scale (Section 4.1), macro‐scale near‐field (Section 4.2), and macro‐scale far‐field (Section 4.3) simulations are shown and discussed.

### 4.1 Micro‐Scale Simulations

**Figure 8:**
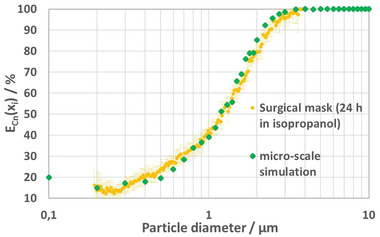
Filtration efficiency of a surgical mask obtained using micro‐scale simulations (green symbols) accounting for the mechanical filtration mechanisms only and using experiments according to Berger et al.^[^ ^26^ ^]^ In the experiments, discharged masks (treatment by isopropanol for 24 h for comparability).

**Figure 9:**
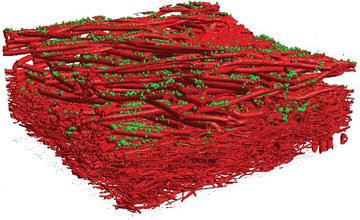
Particles (green) captured by the fibers (red) of a surgical mask as predicted by micro‐scale simulation.

The fractional efficiency and pressure drop obtained by the micro‐scale simulations described in Section 3.1 is shown in **Table** **8** and compared to the experimental results of Berger et al. – exemplarily for the case of a surgical mask – in **Figure** **8**.^[^ ^27^ ^]^ In the experiments, the masks were soaked with isopropanol for 24 h in order to discharge the mask and validate the fractional filtration efficiency obtained by numerical simulation based on the “mechanical” separation mechanisms only (impaction, interception, and diffusion) by comparison to the fractional filtration efficiency measured by Berger et al.^[^ ^27^ ^]^ **Figure** **9** shows the particles (in green) captured by the fibers (red) of the mask.

**Table 8:** Macro‐scale parameters (permeability, porosity) of a surgical mask determined by micro‐scale simulation

Permeability [m^2^]	Porosity [‐]
2.34e^−11^	0.913

It can be seen that the fractional filtration efficiency predicted by numerical simulations and that obtained experimentally are in excellent agreement.

### 4.2 Near‐Field Macro‐Scale Simulation

The pressure drop determined by macro‐scale near field simulations using a surgical and an FFP2 mask is shown in **Table** **9** and validated by comparison to experimental results obtained by Berger et al.^[^ ^27^ ^]^

**Table 9:** Pressure drop at 30 l min^−1^ of macro‐scale near‐field simulations compared to measurements of Berger et al.^[^ ^27^ ^]^

Mask type	Pressure drop Macro‐scale simulation [Pa]	Pressure drop Experiment [Pa]
Surgical mask	27,5	29
FFP2 mask	102	108

In Table 9, it can be seen that the pressure drop measurements correspond well to the pressure drop results of the macro‐scale simulations with deviations of less than 5%. This confirms that the parameters of the micro‐scale simulations are reasonable and can be used in the macro‐scale far‐field simulations for the predictions of particle fates.

### 4.3 Macro‐Scale Far‐Field Simulation

In Section 4.3.1, particle emission by exhaling through the nose and mouth, speaking, and coughing as well as inhalation (Section 4.3.2) are considered. For each scenario, the influence of different mask‐wearing modes is investigated and compared (sealed mask, mask with leakage, and no mask).

#### 4.3.1 Particle Emission

For the emission scenarios exhaling through the nose, exhaling through the mouth, speaking, and coughing, the velocities given in Table 2 are defined as inlet velocity.

##### 4.3.1.1 Velocity Fields

**Figure 10:**
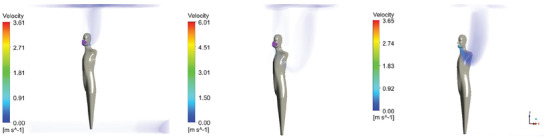
Steady‐state velocity field for exhaling through the nose (sealed mask left, leakage mask middle, and no mask right).

**Figure 11:**
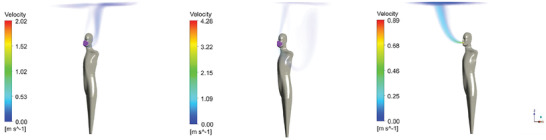
Steady‐state velocity field for exhaling through the mouth (sealed mask left, leakage mask middle, and no mask right).

**Figure 12:**
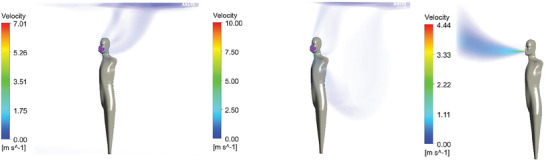
Steady‐state velocity field for speaking (sealed mask left, leakage mask middle, and no mask right).

**Figure 13:**
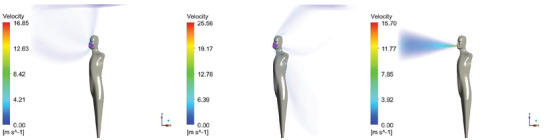
Steady‐state velocity field for coughing (sealed mask left, leakage mask middle, and no mask right).

The results of the steady‐state velocity magnitude of the four emission scenarios are shown in **Figures** **10**, **11**, **12**, **13**.

Figures 10, 11, 12, 13 show that while wearing a mask, the flow is generally mainly directed upwards. This is due to thermal buoyancy induced by the emitted air being warmer than the ambient temperature on the one hand and due to the heat emission of the person on the other hand. Only in the case of coughing with a perfectly fitting (sealed) mask, the velocity of the emitted air is large enough so that the flow is still clearly directed forwards after passing the mask. For the other emission scenarios, the uplift of the flow occurs very close to the head of the person itself. The mask may also act as a barrier that deflects the flow backward. This effect is especially pronounced for the higher emission velocities, specifically in the case of speaking and coughing with a naturally fitted (leaking) mask.

In the leakage case, the flow is strongly directed toward the leakage areas at the bottom and top of the mask. This effect becomes more pronounced for larger velocities. Even the air that exits the leakage area downwards does not reach the ground but is directed upward due to buoyancy forces. In case no mask is worn the flow is initially strongly directed forward. In comparison to the results with mask the uplift of the flow occurs further away from the person because the flow is not deflected by the mask. In the case of coughing, the horizontal velocity is so high that the buoyancy has hardly any influence within the 1.5 m distance from the infected person considered in this study. Close to the bottom, the air is flowing back to the person. This effect becomes especially obvious in the case of exhaling through the nose with a perfectly fitting/sealed mask, see Figure 10, left‐hand side.

##### 4.3.1.2 Particle Fates

**Figure 14:**
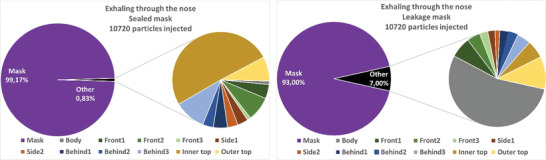
Fates of injected particles for exhaling through the nose (sealed mask left and leakage mask right).

**Figure 15:**
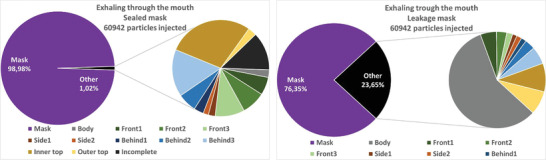
Fates of injected particles for exhaling through the mouth (sealed mask left and leakage mask right).

**Figure 16:**
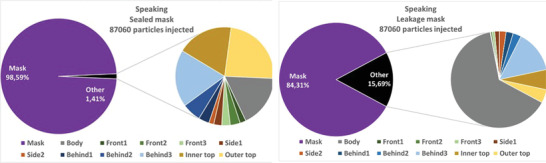
Fates of injected particles for a speaking person (sealed mask left and leakage mask right).

**Figure 17:**
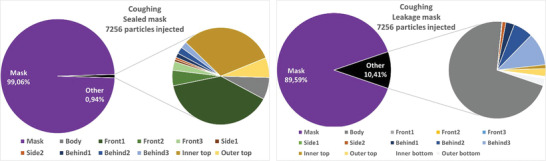
Fates of injected particles for a coughing person (sealed mask left and leakage mask right).

The absolute and relative particle fates are investigated for the different emission scenarios in the following. This means that particles are tracked until they hit a boundary and then counted and summed up. Since most of the particles are captured by the mask, for the cases with mask, the penetrating particles (“other”) are split up in further detail in **Figures** **14**, **15**, **16**, **17** on the right hand sides. The colors of the zones (3x front, 2x side, and 3x behind) are consistent with the initial definition in Figure 5. Absolute particle fates are compared to the case without mask in Section 4.3.1.3.

Again, the mask captures most of the particles. A comparison of the filtration efficiencies for the four emission scenarios is shown in **Table** **10**.

**Table 10:** Comparison of filtration efficiency of sealed mask to leakage mask for different emission scenarios

Emission scenario	Filtration efficiency (mask sealed to face) [%]	Filtration efficiency (leakage mask) [%]
Exhaling through the nose	99.2	93
Exhaling through the mouth	99.0	76.3
Speaking	98.6	84.3
Coughing	99.1	89.6

Table 10 shows that the filtration efficiency of the mask decreases due to leakage depending on the emission scenario. When exhaling through the nose, the efficiency drops to only 93%, while when exhaling through the mouth the efficiency decreases to 76.3%. Since velocities are higher and the percentage of large particles is higher while speaking and coughing, the filtration efficiency is larger in these cases.

Also, it becomes apparent from Figures 14, 15, 16, 17 that leakage leads to the fact that the number of particles hitting a person increases significantly. Thus, regardless of the emission type, the relative number of particles hitting the person is larger than that hitting all other boundaries. This makes sense since the leakage surfaces, and thus the leaking flow, at the bottom and top are directed toward the person itself as shown in Figures 10, 11, 12, 13.

Wearing a sealed mask, the relative number of particles that reach the ceiling is the largest (except for coughing). This is consistent with the velocity fields shown in Section 4.3.1.1. Leakage during coughing causes a significant change. Without leakage, the relative number of particles reaching the front zones (green color) is almost halved. If there is leakage the particles are clearly directed to the back. This is consistent with the velocity field shown in Figure 13.

##### 4.3.1.3 Comparison to the “no mask” Case

**Figure 18:**
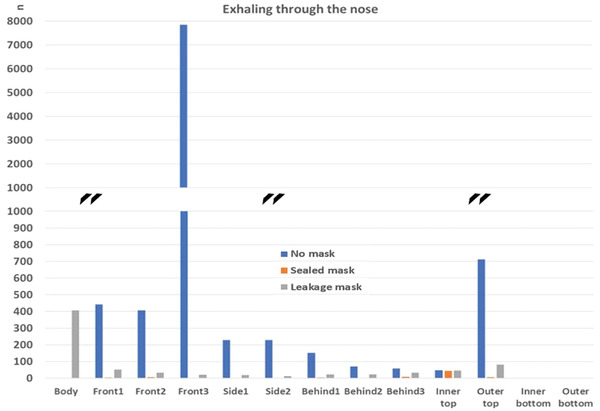
Particle fates outside the mask for the scenario “exhaling through the nose”.

**Figure 19:**
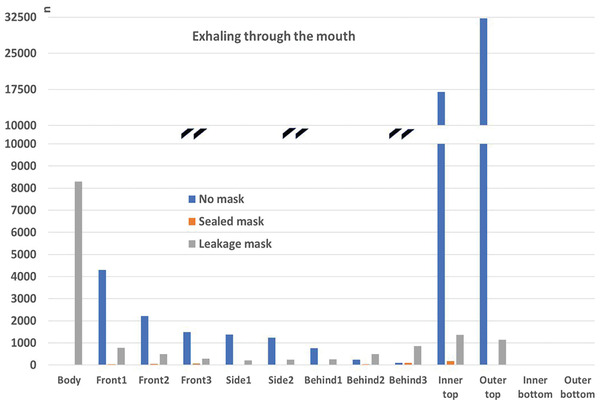
Particle fates outside the mask for the scenario “exhaling through the mouth”.

**Figure 20:**
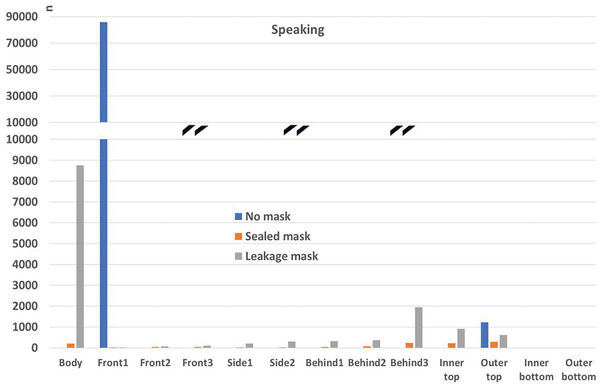
Particle fates outside the mask for the scenario “speaking”.

**Figure 21:**
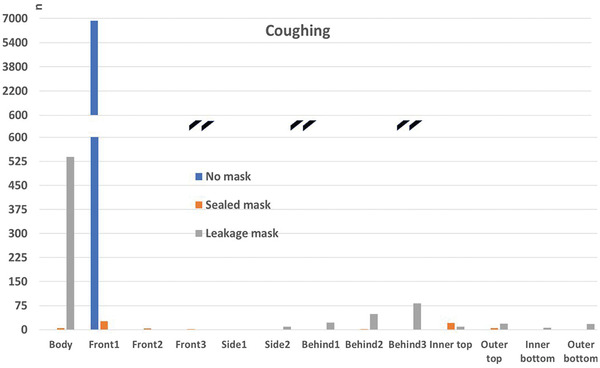
Particle fates outside the mask for the scenario “coughing”.

In the following, the absolute particle fates for the cases with mask (sealed to face), mask with leakage, and without mask are compared. In order to account for the vastly different particle numbers, the vertical axis is interrupted. This is marked with black double lines (**Figures** **18**, **19**, **20**, **21**).

It can be seen that a mask significantly reduces the number of particles reaching any of the boundaries compared to the case without mask independent of the fit of the mask (“sealed to face” compared to “leakage mask” case). It needs to be stressed, however, that the filtration efficiency without leakage is higher (Table 9). Only the number of particles that reach the person is higher in the “leakage mask” case compared to the case without mask. The reason for this has already been discussed in Section 4.3.1.2.

#### 4.3.2 Inhalation

Since inhalation predominantly takes place through the nose, only this case is considered in the following. Therefore, in our model, the velocity magnitude specified in Table 2 is defined at the “inlet”, but the direction is inverted. The particles are injected on planes as described in Section 3.5.

##### Flow Results

**Figure 22:**
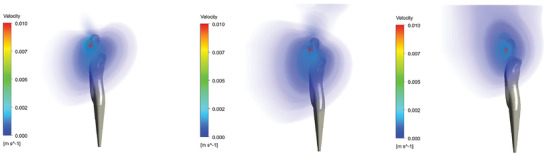
Steady‐state flow solutions for inhalation through the nose (sealed mask left, leakage mask middle, and no mask right).

The steady‐state flow solution for inhalation through the nose with sealed mask, leakage mask, and without mask is shown in **Figure** **22**.

It can be seen that the velocities are already negligibly small at a short distance from the person. In order to be able to visualize the velocities at a distance from the person at all, the maximum velocity is limited to 0.01 m s^−1^.

##### Particle Fates

**Figure 23:**
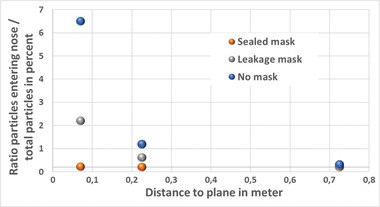
Ratio of particles entering the nose over totally inhaled particles as a function of distance (with mask right, mask with leakage middle, and no mask right).

One main parameter determining infection risk is the number of particles being inhaled. In **Figure** **23**, the ratio of particles that enter the nose over the total number of particles is plotted as a function of the distance from the middle of the domain. This consideration is again related to the different situations with masks, masks with leakage, and no masks.

For all cases, the number of inhaled particles decreases strongly with increasing distance. Almost no particles are inhaled from distances > 0.7 m. Wearing a mask significantly reduces the number of particles that enter the nose although leakage significantly affects the ratio compared to a mask without leakage.

## 5 Summary and Outlook

The purpose of this work was to investigate the far‐field protection effect of masks (“particle fates”) depending on the emission scenario (breathing, speaking, and coughing) and the compliance of mask‐wearing (perfectly fitting mask, naturally fitted mask, and no mask). In addition, a workflow was developed on how to carry through parameters from microCT scans of mask filter media over micro‐scale simulations, near‐field macro‐scale simulations to macro‐scale far‐field simulations.

Fractional filtration efficiency of discharged masks determined by micro‐scale simulations on the CAD geometry of the fibrous porous medium obtained by microCT scan was in excellent agreement with experimental results. The pressure drop obtained by macro‐scale near‐field simulations accounting for the shape of the mask is shown to be in good agreement with experimental data (deviation < 5%). For the far‐field simulations, we considered both the steady‐state flow and the particle transport in an open room. It can be seen that wearing a mask has a significant effect on the flow field and also on the particle fates. Particularly, the flow is strongly deflected by a mask and the direction of the flow is even directed backward when particles are emitted with high velocities during speaking and coughing. If the mask is not fitting perfectly, the major part of the flow is directed to the leakage areas and thus, the overall filtration efficiency is significantly reduced. Nevertheless, even a mask with leakage still strongly reduces the dispersion of particles compared to exhalation without a mask. For inhalation, again, a mask significantly reduces the entry of particles into the nose while leakage again limits the filtration efficiency.

Using the proposed modeling and simulation workflow allows for the optimization of masks in the future. This concerns both the shape of the mask and its effect on pressure drop in near‐field simulations as well as the far‐field effect of the mask and an improved material selection. Furthermore, far‐field simulations taking into account the presence of two or more persons in a room will be carried through in the future, also considering the ejection angle of respiration. Finally, particle fates in outdoor environments will be studied where relative humidity, different temperatures, and wind conditions will influence particle transport in the far field.

## Conflict of Interest

The authors declare no conflict of interest.

## Data Availability

The data that support the findings of this study are available from the corresponding author upon reasonable request.
